# Ergebnisse der Sicca-Forschungsförderung 2016

**DOI:** 10.1007/s00347-020-01163-w

**Published:** 2020-07-07

**Authors:** A. Musayeva, A. Gericke, F. Jäger, F. Paulsen, M. Braun, B. Fabry, R. Braun, D. Pauly, C. Holtmann, G. Geerling, Sicca-Preis-Jury, Gerd Geerling, David Goldblum, Jutta Horwath-Winter, Christina Jacobi, Reinhard Kaden, Thomas Kaercher, Elisabeth Messmer, Friedrich Paulsen, Johannes Schwarzkopff, Manfred Zierhut

**Affiliations:** 1 Augenklinik und Poliklinik, Klinikum der Johannes-Gutenberg-Universität Mainz, Mainz, Deutschland; 2 Institut für Funktionelle und Klinische Anatomie, FAU Erlangen-Nürnberg, Erlangen, Deutschland; 3 Institut für Physik der kondensierten Materie, FAU Erlangen-Nürnberg, Erlangen, Deutschland; 4 Klinik und Poliklinik für Augenheilkunde, Universitätsklinikum Regensburg, Regensburg, Deutschland; 5 Klinik für Augenheilkunde, Universitätsklinikum Düsseldorf, Moorenstr. 5, 40225 Düsseldorf, Deutschland

**Keywords:** Muskarinrezeptor, Harnstoff, Tränenfilmstabilität, Graft-versus-Host-Disease, Lidspannung, Muscarinic receptor, Urea, Tear film stability, Graft versus host disease, Eyelid tension

## Abstract

Der Sicca-Förderpreis unterstützt die Entwicklung wissenschaftlicher Arbeiten zu Pathogenese, Diagnostik und Therapie des trockenen Auges und Augenoberflächenerkrankungen. Er wird nach befristeter Ausschreibung im deutschsprachigen Raum, schriftlicher Antragstellung und Preisträgerauswahl nach Begutachtung durch ein Jurorengremium aus grundlagen- und klinisch-wissenschaftlich arbeitenden Augenärzten vergeben. In diesem Beitrag werden beispielhaft die Ergebnisse geförderter Projekte des Sicca-Förderpreises 2016 kursorisch dargestellt, deren Ergebnisse im Rahmen der Augenärztliche Akademie Deutschland 2019 vorgestellt wurden, und damit ein Einblick in die aktuellen wissenschaftlichen Entwicklungen skizziert. Dabei wird die Rolle der Muskarinrezeptoren sowie jene des Harnstoffs in der Pathogenese des trockenen Auges ebenso beleuchtet wie die (fehlende) Korrelation der Tränenfilminstabilität, -viskosität und Oberflächenspannung. Wegweisend sind auch ein Projekt zur Frühdetektion der okulären Beteiligung bei der Graft-versus-Host-Disease und der Gedanke, eine Meibom-Drüsen-Dysfunktion mit lidchirurgischen Techniken zu behandeln. Die skizzierten Projekte stellen das Potenzial für weitere substanzielle Entwicklungen zu Verständnis, Diagnostik und Therapie des trockenen Auges dar. Ihre langfristige klinische Relevanz muss jedoch noch etabliert werden.

## Abstract (en)

The *Sicca-Förderpreis* (Dry Eye Award) supports the development of scientific research on the pathogenesis, diagnostics, and treatment of dry eye and ocular surface diseases. It is awarded after a limited call for proposals in German-speaking countries, written application and selection of the award winner after evaluation by a jury of ophthalmologists working in basic and clinical science. In this article examples of the results of funded projects of the *Sicca-Förderpreis 2016* are cursorily described, which were presented at the Ophthalmological Academy of Germany 2019 (Augenärztliche Akademie Deutschland 2019) and therefore provide an insight into current scientific developments. The role of muscarinic receptors and those of urea in the pathogenesis of dry eye as well as the (missing) correlation of tear film stability, viscosity and surface tension are highlighted. A project on the early detection of ocular involvement in graft versus host disease and the idea of treating meibomian gland dysfunction with eyelid surgery techniques are also groundbreaking. The outlined projects represent the potential for further substantial developments in the understanding, diagnostics and treatment of dry eye; however, their long-term clinical relevance still needs to be established.

Der Sicca-Förderpreis des Ressort Trockenes Auge und Oberflächenerkrankungen im Berufsverband der Augenärzte wird im Jahr 2020 zum 25. Mal auf der Tagung der Deutschen Ophthalmologischen Gesellschaft (DOG) verliehen. Er wurde 1995 begründet von H. Brewitt und fördert wissenschaftliche Arbeiten zu Pathogenese, Diagnostik und Therapie des trockenen Auges und Erkrankungen der Augenoberfläche. Seiner Vergabe gehen eine jährliche, befristete Ausschreibung im deutschsprachigen Raum, eine schriftliche Antragstellung und eine Preisträgerauswahl nach Begutachtung durch ein Jurorengremium aus grundlagen- und klinisch-wissenschaftlich arbeitenden Augenärzten aus Klinik und Praxis voraus.

Lange Zeit wurde die quantitative und qualitative Relevanz dieses Arbeitsgebietes in Deutschland unterschätzt. Nicht zuletzt dank der mittlerweile jahrzehntelangen Förderarbeit hat die Zahl von Arbeitsgruppen, die sich mit dem Gebiet der Ausschreibung beschäftigen, stark zugenommen und ist heute wissenschaftlich und in Form von „Sicca-Sprechstunden“ auch zunehmend klinisch in der deutschen Augenheilkunde verankert worden. In diesem Beitrag werden beispielhaft die Ergebnisse geförderter Projekte des Sicca-Förderpreises 2016, die jeweils 3 Jahre nach der Vergabe im Rahmen eines Ergebnissymposiums berichtet werden, kursorisch dargestellt und damit ein Überblick über die aktuellen wissenschaftlichen Entwicklungen skizziert.

## Grundlagenwissenschaftliche Projekte

### Rolle des M_3_-Acetylcholinrezeptors bei der Tränensekretion

Muskarinische Acetylcholinrezeptoren sind an der neuronalen Kontrolle der Tränenflüssigkeitssekretion beteiligt [1]. In experimentellen Studien werden daher Muskarinrezeptorantagonisten häufig verwendet, um ein trockenes Auge zu induzieren [2, 3]. Fünf Subtypen (M_1_–M_5_) muskarinischer Acetylcholinrezeptoren sind bekannt [4]. Jedoch ist noch nicht genau geklärt, welche Subtypen an der Kontrolle der Tränensekretion beteiligt sind.

Basierend auf Studien an Tränendrüsengewebe verschiedener Spezies unter Verwendung subtypselektiver Muskarinrezeptorliganden und Antikörper, wird eine Beteiligung des M_3_-Rezeptors an der Tränenflüssigkeitssekretion vermutet [5, 6]. Zudem wurden im Blutserum von Patienten mit Sjögren-Syndrom Autoantikörper gegen den M_3_-Rezeptorsubtyp nachgewiesen [7]. Es ist jedoch zu beachten, dass sowohl die Selektivität konventioneller pharmakologischer Liganden als auch von gegen einzelne Muskarinrezeptorsubtypen gerichteten Antikörpern eingeschränkt ist [4, 8, 9].

Musayeva et al. untersuchten daher die Hypothese, ob das Fehlen des M_3_-Rezeptors am genetisch modifizierten Mausmodell (M_3_R^−/−^) ein trockenes Auge induziert. Die Versuche beinhalteten eine Quantifizierung der Tränenproduktion, eine Untersuchung der Epithelbeschaffenheit mittels Fluoreszeinfärbung, die Bestimmung der gesamten Hornhaut- und Hornhautepitheldicke sowie der epithelialen Proliferationsrate an histologischen Hornhautschnitten und die Quantifizierung proinflammatorischer Zytokine mittels quantitativer PCR.

**Figure 1:**
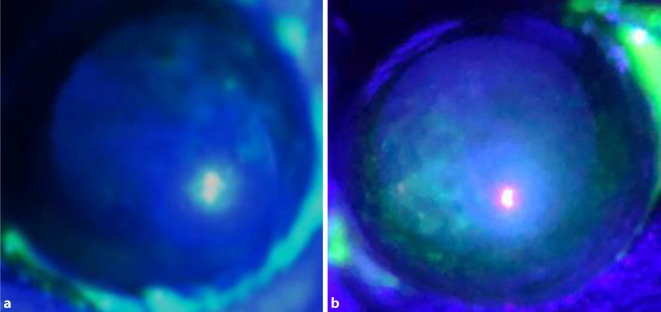

Es zeigte sich, dass die Tränenflüssigkeitsproduktion in M_3_R^−/−^-Mäusen gegenüber den Wildtypkontrollen deutlich reduziert ist. Zudem wiesen M_3_R^−/−^-Mäuse deutliche punktförmige korneale Epitheldefekte auf (Abb. 1), die mit einer Epithelverdünnung und einer reduzierten Proliferationsrate der Epithelzellen vergesellschaftet waren. Des Weiteren wiesen M_3_R^−/−^-Mäuse sowohl in der Hornhaut als auch in der Bindehaut eine erhöhte Expression proinflammatorischer Zytokine auf.

Die Ergebnisse sprechen für eine Beteiligung des M_3_-Rezeptors an der Tränenflüssigkeitssekretion und für die Entwicklung eines trockenen Auges bei den Tieren auch ohne zusätzliche pathophysiologische Reize. Der Vorteil dieses Tiermodells für weitere Studien am trockenen Auge besteht in der Verzichtbarkeit auf wiederholte Applikationen von Pharmaka, wie es bisher zur experimentellen Induktion eines trockenen Auges im Tiermodell nötig war [10, 11]. Somit ist das Mausmodell für Langzeitstudien gut geeignet, um altersabhängige Veränderungen im Rahmen des trockenen Auges zu untersuchen. Ein weiterer Vorteil besteht darin, dass mit dem Modell die Rolle eines einzelnen Muskarinrezeptorsubtyps bei der Pathophysiologie des trockenen Auges untersucht werden kann. Da die verschiedenen Muskarinrezeptorsubtypen an einer Vielzahl von Prozessen im Auge und außerhalb des Auges beteiligt sind, ist entsprechend auch das Nebenwirkungsprofil von nicht-subtypselektiven Muskarinrezeptorliganden, wie Pilocarpin oder Atropin, groß [12–14]. Medikamente, die selektiv den M_3_-Rezeptor aktivieren, könnten künftig ein klinisch wirksames Mittel mit einem verringerten Nebenwirkungsprofil gegen das trockene Auge darstellen.

### Bedeutung von Harnstofftransportern

Harnstoff ist ein Stoffwechselendprodukt von Stickstoffverbindungen wie Aminosäuren (Harnstoffzyklus) und stellt einen festen Bestandteil der Tränenflüssigkeit dar [15]. Dabei besitzt der Metabolit einen stark hydrophilen Charakter, wodurch in einer Vielzahl von Organen, wie z. B. der Niere, der gerichtete Transport von Wasser von der lokalen Harnstoffkonzentration in benachbarten Geweben abhängig ist [16]. Harnstoff ist ein polares Molekül mit entsprechend geringer Membranpermeabilität, dessen zügige Passage über die membranöse Lipiddoppelschicht durch spezifische Transportproteine ermöglicht wird. Bisher sind diese Harnstofftransporter (Ureatransporter [UT]) v. a. aus Niere und Erythrozyten bekannt, ihre Expression wurde aber auch bereits in anderen Organen wie Leber, Herz, Hoden, Haut, Gehirn und dem Kolon detektiert [17]. Es sind 2 unterschiedliche Genfamilien für Ureatransporter beschrieben: SLC14A2 codiert für den Ureatransporter Typ A (UT-A), SLC14A1 entsprechend für den Ureatransporter Typ B (UT-B). Von UT‑A sind verschiedene cDNA-Isoformen bekannt, die als UT-A1, -2, -3, -4 und -5 bezeichnet werden [18].

Enzyme, die Harnstoff herstellen (Arginase 1, Arginase 2, Agmatinase) wurden in den Geweben der Augenoberfläche (Kornea, Konjunktiva) und in der Tränendrüse des Menschen bereits nachgewiesen [19]. Unklar ist jedoch bisher, ob die zellulär synthetisierten Harnstoffmoleküle die Zellmembran frei oder mithilfe ihrer spezifischen Transportproteine (Ureatransporter) passieren. In der vorliegenden Arbeit von Jäger et al. wurde dieser Frage nachgegangen, indem die Augenoberfläche sowie das tränenbildende System der Spezies Mensch und der Modell-Spezies Maus und Schwein hinsichtlich einer möglichen Harnstofftransporterexpression untersucht wurden.

**Figure 2:**
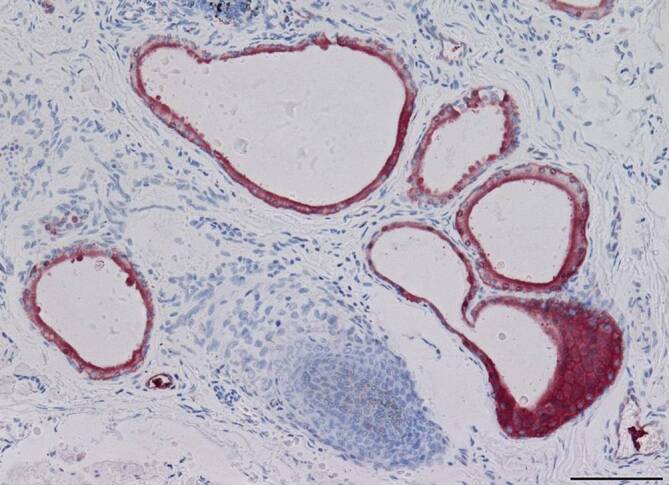

Mittels immunhistochemischer Lokalisationsstudien wurden folgende Gewebe auf eine Expression der Ureatransporter A und B auf Proteinebene untersucht: Kornea, Konjunktiva, Meibom-Drüse, Moll-Drüse (Abb. 2), Zeis-Drüse und Tränendrüse. Immunhistochemisch ließen sich die verschiedenen Isoformen des Typ-A-Transportproteins nicht unterscheiden. Dazu wurde das Verfahren der RT-PCR (reverse Transkriptase-PCR) herangezogen. Bei Mensch und Maus konnten damit folgende Gewebe bezüglich einer möglichen Harnstofftransporterexpression auf RNA-Ebene untersucht werden: Kornea, Konjunktiva, Meibom-Drüse und Tränendrüse.

Bei den immunhistochemischen Gewebestudien zeigte sich eine deutliche Antikörperreaktivität gegen UT‑A sowie gegen UT‑B in allen untersuchten Geweben (Mensch, Maus und Schwein). Die weiteren Untersuchungen auf mRNA-Ebene mittels RT-PCR (Mensch und Maus) ergaben ebenfalls eine positive Expression von UT‑B. RNA des Typ-A-Transporters konnte lediglich im Bereich der humanen Tränendrüse gefunden werden. Der Transporter wurde dabei im humanen Gewebe nicht in seine Isoformen aufgeschlüsselt. Bei der Spezies Maus ließ sich eine Expression des Subtyps UT-A3 in allen untersuchten Geweben detektieren. Die anderen UT-A-Isoformen (UT-A1, -2, -5/UT-A4 ist bei der Maus nicht bekannt) konnten nicht nachgewiesen werden.

Die Expression der Harnstofftransporter in den Geweben der Augenoberfläche und des tränenbildenden Systems lässt vermuten, dass ihnen eine wichtige Rolle zur Befeuchtung des Auges mittels Harnstoffsekretion zukommt. Unterstützt wird die These durch die Ergebnisse vorausgegangener Studien, die eine Verbindung von verminderter Harnstoffkonzentration im Tränenfilm und der Ausbildung eines trockenen Auges herstellten [19]. Dabei ist der zugrunde liegende Pathomechanismus dieser Erkrankung noch weitgehend unbekannt. Denkbar wäre, dass eine Störung in der Funktion oder der Expression der Harnstofftransporter im okulären System zur Destabilisierung des Tränenfilms und somit zur Ausbildung eines trockenen Auges führen könnte.

## Klinische Projekte – Diagnostik

### Oberflächenspannung und Viskosität

Das trockene Auge ist grundsätzlich auf eine verminderte Tränenfilmstabilität und Benetzung der Augenoberfläche zurückführen [20]. Die Stabilität eines Flüssigkeitsfilms reduziert sich bei höherer Oberflächenspannung und geringerer Viskosität [21]. Braun et al. untersuchten daher, ob bei Patienten mit trockenem Auge eine pathologisch erhöhte Oberflächenspannung und/oder verminderte Viskosität der Tränenflüssigkeit vorliegen.

**Figure 3:**
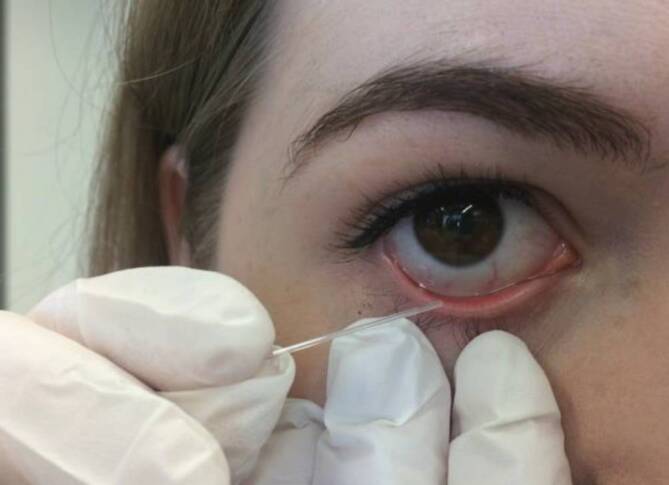

Hierzu wurden 16 gesunde Probanden und 21 Patienten mit moderatem bis schwerem trockenem Auge gemäß Ocular Surface Disease Index (OSDI) und Tränenfilmaufrisszeit (Break-up-Time [BUT]) <5 s, Schirmer-Test I <10 mm und lidkantenparallelen Falten (LIPCOF) Grad >2 untersucht. Unstimulierte Tränenflüssigkeit wurde mithilfe von Glaskapillaren entnommen (Abb. 3), da sich Schirmer-Streifen in Vorversuchen für die Messungen als ungeeignet erwiesen hatten. Die Lagerung der Proben in Eppendorf-Röhrchen bei −20 °C bis zur Messung hatte keinen Einfluss auf die Messergebnisse.

Die Oberflächenspannung wurde anhand der Geometrie eines an einer Kanüle hängenden Tropfens (~40 µl) mit der Pendant-Drop Methode (Easy Drop Shape Analyzer, Krüss) gemessen. Die Oberflächenspannung der im Verhältnis 1:9 mit Phosphat-gepufferter Salzlösung verdünnten Basaltränen von Patienten mit trockenem Auge und von gesunden Probanden zeigte keine signifikanten Unterschiede.

Die Viskosität der Tränenflüssigkeit wurde mittels thermischer Mikrorheologie vermessen, wobei Ø 1 µm fluoreszierende (540/560 nm) Mikropartikel (FluoSpheres, Life Technologies) in 2 µl Tränenflüssigkeit suspendiert wurden. Die Brown-Molekularbewegung der Partikel wurde mit 100 Hz aufgezeichnet und daraus die Viskosität mittels Stokes-Einstein-Formel berechnet.

Die Viskosität der Tränenflüssigkeit bei Sicca-Patienten war im Vergleich zur Kontrollgruppe erhöht. Der Unterschied war jedoch aufgrund einer hohen Streuung statistisch nicht signifikant.

Zusammenfassend zeigen diese Ergebnisse, dass der vorzeitige Tränenabriss bei Patienten mit trockenem Auge nicht auf eine erhöhte Oberflächenspannung oder eine erniedrigte Viskosität der Tränenflüssigkeit zurückgeführt werden kann. Die Grenzflächeneigenschaften des Tränenfilms werden maßgeblich durch Tränenlipide, Peptide und Surfactant-Proteine (SP‑A, -B, -C, -D, -H und -G) reguliert, die die Oberflächenspannung des Tränenfilms reduzieren [22, 23]. Erhöhte Tränenfilmkonzentrationen von Surfactant-Proteinen bei trockenem Auge [23, 24] und – wie in dieser Studie gezeigt – ihre hohe Potenz selbst bei starker Verdünnung erklären die unveränderte Oberflächenspannung. Ein möglicher Einfluss der Lagerung von Tränenfilmflüssigkeit auf die Oberflächenspannung [25] bestätigte sich in der vorliegenden Untersuchung nicht, wie bereits zuvor vermutet wurde [26]. Die Aussagekraft der vorliegenden Untersuchung ist wegen der hohen Genauigkeit, Reproduzierbarkeit und geringen interindividuellen Streuung insbesondere bei der Messung der Oberflächenspannung nicht durch die geringe Probandenzahl limitiert. Allerdings ist von einer möglichen Verdunstung von Tränenflüssigkeit während der häufig zeitintensiven Probenentnahme bei Patienten mit trockenem Auge auszugehen, die durch veränderte Zusammensetzung und Reorganisation der Tränenbestandteile wie Lipide, Proteine und Elektrolyte zu erhöhten Viskositätswerten führen kann [27]. Die in dieser Studie entwickelten neuen Methoden bieten das Potenzial, die Oberflächenspannung und Viskosität von geringsten Probemengen zu messen und die Regulation der Grenzflächeneigenschaften des Tränenfilms besser zu verstehen.

### Frühdetektion der okulären GvHD

Die Graft-versus-Host-Disease (GvHD) ist eine Multisystemerkrankung nach allogener Stammzelltransplantation (aSZT), die durch komplexe immunologische Mechanismen verursacht wird [28]. In 50–80 % der Fälle entwickelt sich eine okuläre Beteiligung, die in einer schweren Form des trockenen Auges resultieren kann [29]. Die Regensburger Arbeitsgruppe um Braun und Pauly versucht daher, Risiko- und Einflussfaktoren sowie frühe Determinanten der okulären GvHD (oGvHD) zu identifizieren.

Hierzu erfolgt fortlaufend die standardisierte ophthalmologische Untersuchung vor und 100 bis 200 Tage sowie in weiteren regelmäßigen Abständen bis zu 5 Jahre nach aSZT. Zusätzliche Untersuchungen erfolgen bei neu aufgetretenen okulären Symptomen und bei Neudiagnose oder Aktivität einer GvHD anderer Organsysteme. Bei jeder klinischen Untersuchung wird der Tränenfilm mittels Schirmer-Streifen gewonnen. Im Tränenfilm werden bekannte Marker für die Pathogenese der GvHD und Komplementfaktoren bestimmt (CXCL‑8, -9, -10, -11, sEGFR, C3a, C5a) [30–33], um deren Potenzial als Biomarker für die Entstehung der oGvHD zu evaluieren. Die Tränenfilmanalyse erfolgt mittels Multiplex-Bead-Assay.

**Figure 4:**
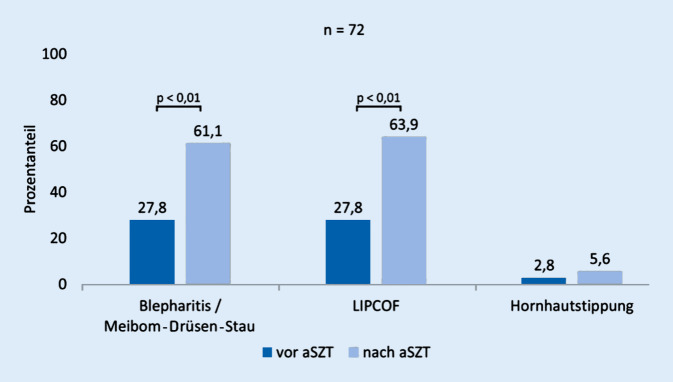

Bislang wurden 95 Patienten im Rahmen der Erstuntersuchung (51,4 ± 34,0 Tage vor aSZT) in die Studie inkludiert und 36 Patienten im Follow-up-Zeitraum 130,8 ± 52,27 Tage nach der aSZT untersucht. An zugrunde liegenden hämatologischen Erkrankungen lagen überwiegend eine akute myeloische Leukämie (*n* = 16) sowie ein myelodysplastisches Syndrom (*n* = 5) vor. Die Stammzellgabe erfolgte überwiegend HLA-ident (*n* = 28), gefolgt von 7 HLA-mismatch- und einer haploidenten aSZT. Zum oben genannten Follow-up-Zeitpunkt hatten bereits 56 % der Patienten eine GvHD anderer Organsysteme erlitten (39 % kutan, 31 % intestinal, 6 % Mundschleimhaut, 3 % Leber). Die Diagnose einer oGvHD wurde bei 11 % der Patienten gestellt. In der ophthalmologischen Untersuchung zeigten sich der Visus sowie der Schirmer-I- und Schirmer-Basis-Wert unverändert. Der Augeninnendruck sowie die BUT waren im Vergleich signifikant verringert, der OSDI-Score hingegen signifikant erhöht (Tab. 1). Zusätzlich konnte ein deutlich vermehrtes Auftreten von Blepharitiden und Meibom-Drüsen-Stau sowie lidkantenparallelen Falten (LIPCOF) festgestellt werden (Abb. 4). Der Hornhautbefund war im Vergleich zur Untersuchung vor der aSZT jedoch noch unverändert. In der Multiplex-Analyse des Tränenfilms zeigte sich ein signifikanter Anstieg von CXCL‑9, das bei der T‑Zell-Aktivierung im Rahmen der chronischen GvHD eine wichtige Rolle spielt [31]. CXCL‑8, -10, -11, sEGFR, C3a und C5a waren zu diesem Zeitpunkt noch unverändert im Vergleich zur Erstuntersuchung. Die Aussagekraft der laborchemischen Untersuchungen ist aufgrund der geringen Probenanzahl und der niedrigen Wiederfindungsraten der Proteine aktuell noch limitiert.

**Table 1:** 

Gesamt *n* = 72 Augen	Vor aSZT (Mittelwert)	Nach aSZT (Mittelwert)	*p*
Visus c.c.	1,0 ± 0,2	0,9 ± 0,2	0,97
Tensio (mm Hg)	15,5 ± 1,9	14,6 ± 2,4	0,01
Schirmer I (mm)	17,5 ± 12,5	15,0 ± 11,4	0,43
Schirmer Basis (mm)	10,8 ± 10,6	10,7 ± 10,0	0,67
Break-up-Time (s)	7,4 ± 4,9	6,2 ± 4,4	0,02
OSDI	9,3 ± 11,5	13,6 ± 13,3	0,03

Anhand der vorliegenden Daten wird evident, dass sich nach einer aSZT schon zu einem sehr frühen Zeitpunkt klinische messbare, wenn auch nicht schwerwiegende Veränderungen der okulären Strukturen zeigen. Die Ursache des Augeninnendruckabfalls nach der aSZT ist zum jetzigen Zeitpunkt noch unklar. Durch Einschluss weiterer Patienten und deren Betreuung über den geplanten Follow-up-Zeitraum über 5 Jahre in dieser Studie werden weitere Erkenntnisse über Risiko- und Einflussfaktoren der oGvHD folgen. Die Analyse des Tränenfilms zeigt bisher noch technische Mängel, welche die Aussagekraft der vorliegenden Ergebnisse einschränkt. Langfristig ist hier die Etablierung einfach und reproduzierbar zu vermessender Biomarker anzustreben, um eine frühzeitigere Diagnose der oGvHD zu ermöglichen [30, 34, 35]. Hierdurch und in Kombination mit den schon früh messbaren oben genannten klinischen Veränderungen könnte eine frühzeitigere therapeutische Intervention ermöglicht werden, um eine weitere irreversible Schädigung der okulären Strukturen zu verhindern. Es wird daher aktuell an der Optimierung des verwendeten Proteinisolationsprotokolls (nach Green-Church et al. [36]) gearbeitet. Aufgrund der leichten Verfügbarkeit und Implementation in die Routinediagnostik soll die Probengewinnung möglichst weiterhin aus Schirmer-Streifen erfolgen [37–39].

## Klinische Projekte – Therapie

### Laterale Zügelplastik bei Meibom-Drüsen-Dysfunktion

Die Meibom-Drüsen liegen in den Tarsalplatten der Augenlider und produzieren den größten Teil der Lipidkomponente des Tränenfilms – das sog. Meibum. Dieses reduziert die Verdunstung der Tränenflüssigkeit, verbessert die Stabilität des Tränenfilms, schützt die Augenoberfläche und ist für eine gute visuelle Funktion essenziell [40]. Die häufigste Form der Meibom-Drüsen-Dysfunktion (MDD) ist die obstruktive Form, die auch im Rahmen von Systemerkrankungen wie Rosazea, seborrhoischer Dermatitis, Atopie oder Psoriasis auftreten kann [41]. Es ist bekannt, dass eine erhöhte Unterlidlaxizität mit Symptomen des trockenen Auges, gemessen mittels OSDI, korreliert [42]. In dieser Arbeit wurde daher geprüft, ob über eine Verbesserung der Unterlidstraffheit mittels lateraler Zügelplastik eine verbesserte Sekretion von Meibum und eine Verringerung der subjektiven wie objektiven Zeichen einer evaporativen Tränenfilmstörung erzielt werden kann.

**Figure 5:**
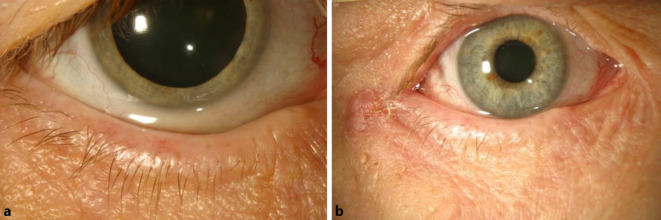

In die prospektive Studie wurden 15 Patienten eingeschlossen, die Symptome eines trockenen Auges (präoperativer OSDI-Score >12), eine verkürzte Tränenfilmaufrisszeit (<10 s), erhöhte Unterlidlaxizität mit positivem Snap-back-Test und positivem Pinch-Test (Abb. 5) sowie keine vorangegangenen lidchirurgischen Eingriffe aufwiesen und in die Operation und die Vor- und Nachuntersuchungen einwilligten. Die Patienten wurden jeweils präoperativ sowie 3 Monate postoperativ standardisiert im Rahmen einer speziellen Sicca-Sprechstunde untersucht.

**Figure 6:**
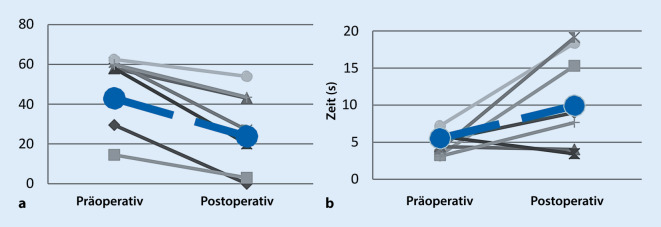

Drei Monate nach dem Eingriff waren die Symptome des trockenen Auges signifikant reduziert (Abb. 6) und die Zahl der funktionellen Meibom-Drüsen (Meibumexpression) sowie die Dicke der Tränenfilmlipidschicht (Lipidinterferometrie) signifikant verbessert. Die nichtinvasive Tränenfilmaufrisszeit (Keratograph) zeigte sich tendenziell verbessert (Abb. 6). Während diese vorläufigen klinischen Ergebnisse aufgrund der geringen Anzahl von Patienten begrenzt sind, stützen sie die Hypothese, dass die Erhöhung der Zugfestigkeit des Augenlids die Funktion der Meibom-Drüsen beeinflussen kann. Um dieses neue Therapiekonzept zu bestätigen, wird aktuell eine umfangreichere Studie durchgeführt.

## Zusammenfassung

Die dargestellten Projekte zeigen die Bandbreite der durch den Sicca-Förderpreis geförderten Arbeiten von grundlagenwissenschaftlichen Arbeiten zur Pathophysiologie über die Frühdiagnose bis hin zur chirurgischen Therapie des trockenen Auges und assoziierter Augenoberflächenerkrankungen. Durch die Identifikation neuer molekularbiologischer Regulationsmechanismen für die Tränensekretion wie auch die Homöostase der Oberflächenepithelien, z. B. in Form von Muskarinrezeptoren oder Harnstoff, ergeben sich langfristig möglicherweise neue Therapieoptionen für diese Volkskrankheit. Der Weg dorthin führt meist über jahr(zehnt)elange klinische Studien. Ob eine Tränenfilminstabilität unabhängig von der Oberflächenspannung oder Viskosität der Tränenflüssigkeit ist, sollte in weiteren Untersuchungen validiert werden, z. B. auch in gut definierten Kohorten wie Patienten mit Graft-versus-Host-Disease. Dies gilt auch für neue, chirurgische Ansätze, die sich noch in der klinischen Praxis bewähren müssen, wie die Tarsalzungenplastik zur Therapie einer Meibom-Drüsen-Dysfunktion. Zumindest für die Zügelplastik darf man allerdings schon in absehbarer Zeit mit weiteren Erfahrungsberichten rechnen, da hier keine weiten Wege über nationale und internationale Zulassungsbehörden erforderlich sind.

## Fazit für die Praxis

- Der Sicca-Förderpreis unterstützt seit 1995 die Entwicklung wissenschaftlicher Arbeiten zur Pathogenese, Diagnostik und Therapie des trockenen Auges und der Augenoberfläche.
- Er wird nach befristeter Ausschreibung im deutschsprachigen Raum, schriftlicher Antragstellung und Begutachtung für Grundlagen- und klinische Projekte vergeben.
- Durch die Identifikation neuer molekularbiologischer Regulationsmechanismen für die Tränensekretion und Homöostase der Oberflächenepithelien ergeben sich neue Therapieoptionen für diese Volkskrankheit.
- Die Translation erfordert für neue pharmakologische wie auch chirurgische Ansätze klinische Studien.
